# *HeatMapViewer*: interactive display of 2D data in biology

**DOI:** 10.12688/f1000research.3-48.v1

**Published:** 2014-02-13

**Authors:** Guy Yachdav, Maximilian Hecht, Metsada Pasmanik-Chor, Adva Yeheskel, Burkhard Rost

**Affiliations:** 1 TUM, Department of Informatics, Bioinformatics & Computational Biology, 5748 Garching/ Munich, Germany; 2 TUM Graduate School of Information Science in Health (GSISH), 85748 Garching/Munich, Germany; 3 Biosof LLC, New York, NY, 10001, USA; 4 Bioinformatics Unit, G.S.W. Faculty of Life Sciences, Tel Aviv University, Tel Aviv, 69978, Israel

## Abstract

**Summary: **The HeatMapViewer is a BioJS component that lays-out and renders two-dimensional (2D) plots or heat maps that are ideally suited to visualize matrix formatted data in biology such as for the display of microarray experiments or the outcome of mutational studies and the study of SNP-like sequence variants. It can be easily integrated into documents and provides a powerful, interactive way to visualize heat maps in web applications. The software uses a scalable graphics technology that adapts the visualization component to any required resolution, a useful feature for a presentation with many different data-points. The component can be applied to present various biological data types. Here, we present two such cases – showing gene expression data and visualizing mutability landscape analysis.

**Availability:** https://github.com/biojs/biojs; http://dx.doi.org/10.5281/zenodo.7706.

## Introduction

Biological data are often organized into matrices in which the rows signify different items of interest (a gene, a subject, a probe or a position in a sequence), while the columns describe different experiments, variations, or samples. Matrices are easy to process by algorithms. In contrast, the details in large matrices are often, at best, challenging for experts who want to “understand” the data. The information in matrices is usually better digested if presented by 3D plots or heat maps. Heat maps are essentially simplified versions of 3D plots that replace the 3rd dimension with color gradients, thereby conveniently displaying the information contained in matrices. Such heat maps allow for easy visual differentiation between high and low values in a matrix.

Such heat maps are, for example, commonly used to display microarray data as they quickly show which genes (rows) are differentially expressed under some conditions (columns). Microarray technologies utilize arrays of probes located on different exons for each gene and can be helpful in determining gene function by measuring transcription and translation levels under certain experimental conditions. The expression values for the differential expression may be presented at the exon level, correlated with protein domains, and may help to decipher a complex gene expression pattern.

Heat maps can also display the effect of point mutations (single amino acid substitutions, or non-synonymous Single Nucleotide Polymorphisms – nsSNPs). Through the application of methods that predict the impact of mutations ^1– 4^ we can expand from the view of single variants to the level of sketching the entire *mutability landscape* ^5^. This *mutability landscape* is defined by the impact of substituting every residue at each position in a protein by each of the 19 non-native amino acids. The resulting predictions can then be shown in a heat map in order to visualize the impact of each substitution. Regions where mutations have a high average effect (i.e. where almost every substitution is predicted to alter protein function) are especially interesting as these are likely to be of particular and direct importance for protein function.

We developed *HeatMapViewer* as a BioJS component that can easily be used, reused and, if needed, extended to display matrix data. BioJS ^6^ is an open source JavaScript library of components for visualization of biological data on the web. As a JavaScript component, the *HeatMapViewer* is very flexible, interactive and webready. Previous libraries generating graphical HeatMaps render either static images ^7^ or are highly specialized and cannot be reused ^8^. To the best of our knowledge, this is the first client-side modular component to visualize matrices that can be integrated into other web applications in a standard manner.

## The *HeatMapViewer* component

*HeatMapViewer* uses the D3 ^9^ JavaScript library to render Scalable Vector Graphics (SVG) objects. SVG technology is now standardized and native to modern web browsers (e.g. Mozilla, Chrome, Safari). The component accepts a simple JSON object containing the data matrix that will be rendered. A secondary JSON object contains configuration directions such as the target DIV element onto which the component will be rendered, the data range to be shown, the color scheme to be used for the component, the size of the canvas showing the component and the minimum cell size (by default these last two options can be computed automatically).

The *HeatMapViewer* component automatically renders a heat map based on the input data object and the pre-set color-scheme. Positioning and layout are automatically calculated given the available browser window size. If presenting the entire heat map requires individual cells to be smaller than a given threshold, a secondary panel is automatically rendered to show a zoomed-in version of a local segment in the heat map. This zoom-in panel is presented right under the main heat map panel. The labels for the X-axis and Y-axis are laid out above the top row and next to the left column. The component provides a user control in the form of a frame that can be dragged along the main heat map to determine which area of the heat map should appear in the zoom-in panel. Additionally, a scale bar is presented to show the value ranges and which colors correspond to those values. Finally, each cell in the heat map is associated with a mouse-over event that pops-up tooltips showing the data-value of the cell.

The *HeatMapViewer* component can be obtained from the BioJS registry at https://github.com/biojs/biojs. For users wishing to test the component’s capabilities to generate heat map plots for their data without downloading and installing the component, we have set up a server: http://www.rostlab.org/services/heatmap-viewer. The server allows users to upload their data in Comma Separated Values (CSV) format and then renders a heat map on the screen. The server also allows exporting the resulting graphics rendering it into an image.

## Application use-cases and examples

### Eye disease Retinitis Pigmentosa (RP)

The rhodopsin gene encodes a protein of the outer photoreceptor segment that is essential for the visual transduction cascade. Since 1989, many mutations in the rhodopsin gene have been found to be involved in the eye disease Retinitis Pigmentosa (also known as Retinopathia pigmentosa or simply RP ^10^). RP is a hereditary disease causing retinal degeneration and thereby destroying photoreceptors; this results in severe vision impairment or even blindness.

A typical study of such a hereditary disease might begin with a protocol as follows. According to the UCSC genome browser ^11^, human rhodopsin (RHO, RefSeq: NM_000539.3) consists of 5 exons (located on chr3:129,247,482-129,254,187). The total gene length is 6706 bps (base pairs/nucleotides). The coding region (chr3:129,247,577-129,252,561; i.e. extending over 4985 bps), is translated into a gene-product/protein with 348 residues (UniProt identifier: P08100 ^12^, SwissProt identifier: OPSD_HUMAN ^13^). This protein has a single large domain (Pfam identifier: PF00001 ^14^) that is dominated by a "standard" 7-transmembrane receptor region (rhodopsin family), which spans most of the coding region (residues 55 - 306). The human rhodopsin is highly expressed in the heart, liver, skeletal muscle, thyroid and the eye retina.

### Viewing gene expression data

**Figure 1.**
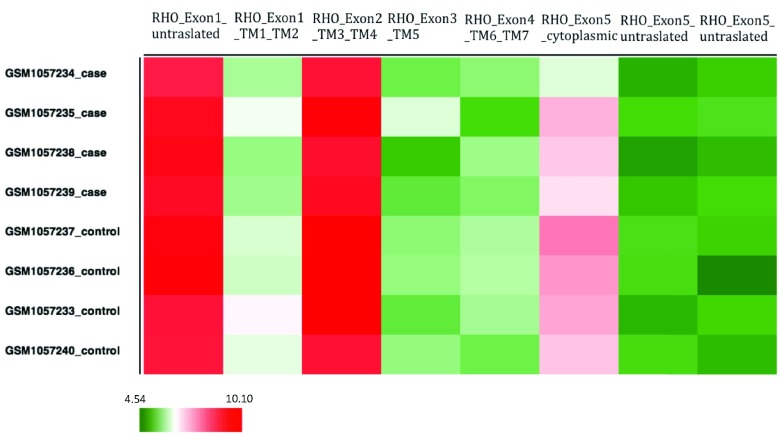
*HeatMapViewer* component visualization of microarray expression experiment (Korir *et al.* 2012; GSE43134). In this experiment, a mutation in a splicing factor that causes Retinitis Pigmentosa (RP) was shown to have an effect on mRNA splicing. Moreover, mutations in the rod photoreceptor-specific protein rhodopsin (RHO) are known to cause RP. Log2 expression values for the 8 probes of human RHO were obtained and located to each of its 5 exons and the 7 trans-membrane (TM) regions (columns). It is interesting to note that the different probes (located on the various regions of RHO), are differentially expressed (high expression colored red and low expression in green). Moreover, we can observe that some RHO probes are expressed differently in the control than in the treatment (case, rows). These results may indicate the effect of the mutated splicing factor on RHO gene in RP disease.

It is interesting to locate the array probes intensities on the various protein domain regions. We map the expression profiles of the RHO (from GEO43134) to the structural protein regions through visualization with the *HeatMapViewer* component ( Figure 1). The different experimental conditions are presented on the rows, while the probes for the RHO gene are shown on the columns, annotated with exon and trans-membrane (TM) location. Probes with high expression are marked in red; those with low expression are colored green. The differences in color of the same probe along the different conditions provides useful information concerning the expression intensity of the various probes, and possible variations in alternative splicing patterns and region conservation across the different samples.

### Predicted protein mutability landscape

**Figure 2.**
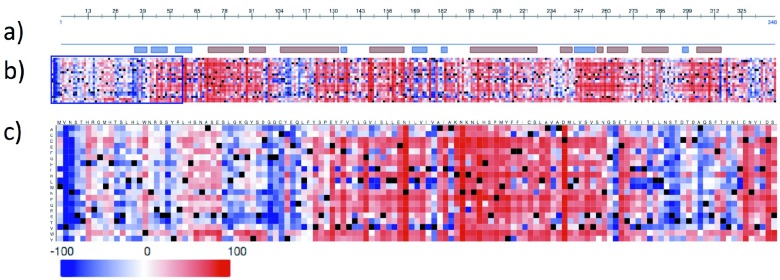
The *HeatMapViewer* component displays the mutability landscape of OPSD_HUMAN. Panel **a**) sketches the secondary structure (helices in red, beta strands in blue). Panel **b**) shows the predictions of effects for each amino acid substitution. Effects are depicted as color intensities ranging from dark blue (high probability of no or little effect) over white (effect can not be predicted or only with very low reliability) to dark red (high probability of strong effects). Black depicts wildtype residues. The blue box marks the zoomed-in region shown in panel **c**).

Since RP is caused by mutations in the rhodopsin gene, researchers have extensively investigated different variations of the gene. Thus, up to now over 100 mutations have been identified and associated with RP. More generally: single nucleotide variations constitute most of the genetic variation among humans and therefore play an important role when studying hereditary diseases or differential drug response. In this context, we show another possible application of the *HeatMapViewer*, again using the 7TM human rhodopsin (SwissProt identifier: OPSD_HUMAN ^13^). The *HeatMapViewer* provides a fast and easy way to represent high dimensional data in a visually comprehensible way that immediately conveys where mutations are likely to be deleterious. Without using a tool such as the *HeatMapViewer*, we could hardly obtain an overview of the protein mutability landscape ^5^. Mutability landscape studies involve predicting the effect of all possible nsSNPs through computational methods, visualizing the predictions in heat maps and cross-linking these predictions with additional sources of information (such as secondary structure, active sites and correlated mutational behavior). Such regions might highlight important aspects of RP. To this end, heat maps ( Figure 2b, 2c) can easily distinguish between low effect regions (represented in blue) and high effect regions (represented in red) while additional information (such as the secondary structure; Figure 2a) can simply be over-laid. These two components already perfectly convey the information that high effect regions are mainly found in the transmembrane helices and in close proximity of the binding sites. Displaying this simple fact without a heat map would be daunting due to the high dimensionality of the underlying data.

## Conclusions

The *HeatMapViewer* component provides a new, powerful way to generate and display matrix data in web presentations and in publications. The use of scalable graphics enables the rendering of high-resolution images as the interactive nature of the component permits those graphics to be scaled on-demand. Furthermore the component can be applied to different cases highlighting various points of interest from gene expression levels to the effects of mutability on protein function. Finally, to make the *HeatMapViewer* component widely accessible, we set up a public web server to which users can upload their matrix data and use the resulting code to show an interactive heat map.

## Software availability

Zenodo: HeatMap Viewer, doi: 10.5281/zenodo.7706 ^15^

GitHub: BioJS, https://github.com/biojs/biojs
