# Supplementary material for: HeatMapViewer: interactive display of 2D data in biology
Source: F1000Res. 2014 Feb 13;3:48. [Version 1] doi: 10.12688/f1000research.3-48.v1 (PMC4023661; doi:10.12688/f1000research.3-48.v1)
Supplement: Peer review history

# Peer review: Referee response for version 1

**Published:** 2014-03-05

**Authors:** John Ison

**Affiliations:** 1 EMBL European Bioinformatics Institute, Hinxton, Cambridge, UK

The article is very well written and tells a nice story, with compelling real-world examples. The software should have scientific impact by providing a convenient overview of matrix data, along the lines already suggested by the authors, and certainly by saving other scientist/developers the need to develop such functionality. 

The software fulfills a need for an easy to use widget for heatmap rendering and there is no doubt that this is a valuable contribution to the BioJS collection. However, I believe It would help many readers if the article placed the software in the broader context of similar offerings, so it would be good if the article enumerated these, perhaps as some sort of "feature matrix"?  

I appreciated the test server very much and encourage the authors to support this service in the future and further develop the functionality.  Please note that examples of the CSV format are needed (at least I could not find them).

I have read this submission. I believe that I have an appropriate level of expertise to confirm that it is of an acceptable scientific standard.

# Peer review: Referee response for version 1

**Published:** 2014-02-27

**Authors:** Jordi Deu-Pons

**Affiliations:** 1 Biomedical Genomics Group, University Pompeu Fabra (UPF), Barcelona, Spain

This is an interesting article and piece of software. I think it contributes towards further alternatives to easily visualize high dimensionality data on the web. It’s simple and easy to embed into other web frameworks or applications.

***Minor revisions***

**a) About the software**

1 - CSV format. It was hard to guess the expected format. The authors need to add a syntax description of the CSV format at the help page.

2 - Simple HTML example. It will be easy to test HeatmapViewer (HmV) if you add a simple downloadable example file with the minimum required HTML-JavaScript to set up a HmV (without all the CSV import code).

3 - Color scale. HmV only implements a simple three point linear color scale. For me this is the major weakness of HmV. It will be very convenient that in the next HmV release the user can give as a parameter a function that manages the score to color conversion.

**b) About the paper**

1 - Introduction (4th paragraph): There are many alternatives to explore a dataset using heat maps. The author only cites two and it’s not clear if you refer to “JavaScript” or “web” alternatives. I think that you have to emphasize the strengths of HmV in comparison to other alternatives (in my opinion, one strength is that it is a good lightweight alternative to embed heat maps in a web report). Example of alternatives that I know of (but I’m sure that there are many more) are:

- http://www.broadinstitute.org/gsea (desktop)
- http://jheatmap.github.io/jheatmap/ (website)
- http://www.gitools.org/ (desktop)
- http://blog.nextgenetics.net/demo/entry0044/ (website)
- http://docs.scipy.org/doc/numpy/reference/generated/numpy.histogram2d.html (python)
- http://matplotlib.org/api/pyplot_api.html (python)

2 - Predicted protein mutability landscape: The authors say: “Without using a tool such as the HeatmapViewer, we could hardly obtain an overview of the protein mutability landscape”. This paragraph seems to suggest that you can explore the data with HmV. I think that HmV is a good tool to report your data, but not to explore it.

3 - Conclusions: The authors say: “... provides a new, powerful way to generate and display matrix data in web presentations and in publications.” To use heat maps in web presentations and publications is nothing new. I think that HmV makes it easier and user-friendly, but it’s not new.

I have read this submission. I believe that I have an appropriate level of expertise to confirm that it is of an acceptable scientific standard.
